# How Visual Style Shapes Tourism Advertising Effectiveness: Eye-Tracking Insights into Traditional and Modern Chinese Ink Paintings

**DOI:** 10.3390/jemr18050042

**Published:** 2025-09-12

**Authors:** Fulong Liu, Xiheng Shao, Zhengwei Tao, Nurul Hanim Md Romainoor, Mohammad Khizal Mohamed Saat

**Affiliations:** 1 School of Arts, Universiti Sains Malaysia, Gelugor 11700, Malaysiahanim.romainoor@usm.my (N.H.M.R.); 2 Centre for Instructional Technology and Multimedia, Universiti Sains Malaysia, Gelugor 11700, Malaysia; shaoshao@student.usm.my; 3 School of Fine Arts and Design, Hechi University, Hechi 546300, China

**Keywords:** advertising, eye movement, traditional Chinese painting, aesthetic evaluation, tourism intention

## Abstract

This study investigates how traditional versus modern Chinese ink painting styles in tourism advertisements affect viewers’ visual attention, aesthetic evaluations, and tourism intentions. Using eye-tracking experiments combined with surveys and interviews, the researchers conducted a mixed-design experiment with 80 Chinese college students. Results indicate that traditional ink-style advertisements attracted longer total fixation durations, higher aesthetic evaluations, and stronger cultural resonance in natural landscape contexts, while modern ink-style advertisements captured initial attention more quickly and performed better aesthetically in urban settings. Qualitative analyses further revealed cultural familiarity and aesthetic resonance underpinning preferences for traditional style, whereas modern style mainly attracted attention through novelty and creativity. These findings expand Cultural Schema Theory and the aesthetic processing model within advertising research, suggesting practical strategies for tourism advertising to match visual styles appropriately with destination types and audience characteristics to enhance promotional effectiveness.

## 1. Introduction

In digital media environments, tourism advertisements rely on visual cues to capture attention, construct destination image, and influence travel intention. While ink aesthetics are increasingly used to convey cultural meanings, the mechanisms through which different ink styles (traditional vs. modern) shape visual processing and persuasion remain underspecified.

We advance an integrated theoretical framework built on four pillars:

(1) Aesthetic processing and processing fluency: Aesthetic experience unfolds from early perception and attention to meaning formation and appraisal; when visual features align with internal schemas, fluency increases, enhancing liking and evaluation.
(2) Style–content congruity (match-up): Messages are more persuasive when form fits semantics/context, improving comprehension and memory.
(3) Environmental psychology (natural vs. urban): Attention Restoration Theory and Psychoevolutionary Theory suggest natural scenes elicit harmony/low arousal and restoration, whereas urban scenes afford stimulation and novelty seeking.
(4) Cultural schemas and cross-cultural perception: Cultural background shapes gaze allocation and style interpretation, moderating the effectiveness of stylistic cues across audiences.

From this, we propose a style–context congruity account. Traditional ink (restraint, graded tonality, negative space) should better fit natural destinations, yielding greater fluency and positive evaluations, while modern ink (contrast, geometric structure, dynamism) should better fit urban destinations, heightening attention and interest.

Congruity should manifest in process (eye-tracking) metrics and translate into outcomes (aesthetic evaluation, travel intention). Incongruity may reduce fluency and persuasion, unless reframed as creative novelty for certain segments.

Despite valuable insights into visual communication in tourism advertising, two gaps persist:

(1) Insufficient style coverage and cultural evidence: Systematic, mechanism-focused comparisons of culturally artistic styles—especially traditional ink—are scarce; differences between traditional and modern ink along the attention–evaluation–intention pathway remain unclear.
(2) Methodological siloing: Most studies rely on a single method (e.g., surveys), lacking an integrated view that combines objective process data (eye movements) with subjective judgments, obscuring the pathway from visual stimuli to psychological and behavioral outcomes.

We therefore adopt a mixed-method design (eye-tracking + subjective evaluations) to compare traditional and modern ink styles across natural/urban destinations, providing theoretical and empirical support for the mechanism of style, context congruity, attention, evaluation/intention.

Based on the above research gaps, this study selected young people in China to explore the following specific research questions:

RQ 1: How do tourism ads using different ink painting styles differ in the allocation of visual attention?

RQ 2: Does the difference in ink painting style in ads lead to changes in audiences’ aesthetic evaluations and tourism intentions?

RQ 3: How do viewers subjectively perceive and interpret the impressions brought by ads with different ink styles?

## 2. Literature Review

### 2.1. Visual Attention and Aesthetic Processing Mechanisms in Tourism Advertising

Visual elements in tourism advertisements steer attention, shape rapid impressions, and influence intentions [1]. Viewers form an overall “gist” of an image within a short time, a process tightly linked to how attention is allocated [2]. Aesthetic experience is not merely preference expression but a multi-stage perceptual–cognitive process; the five-stage model explains the transition from initial input to cognitive appraisal and aesthetic judgment, highlighting the interplay of sensory evidence and interpretation at each stage [3]. Each stage reflects an interaction between sensory input and cognitive interpretation. This model emphasizes that attention responses triggered by visual stimuli, together with higher-level meaning interpretation, jointly determine the emergence of aesthetic experience. From the perspective of user interface design, scholars have proposed two dimensions of aesthetics: classical aesthetics (order and harmony) and expressive aesthetics (novelty and emotional arousal) [4]. Classical aesthetics stress the pleasure derived from simple layouts and clear structure, whereas expressive aesthetics highlight uniquely creative design attributes that evoke emotional resonance. This classification provides a theoretical foundation for understanding the visual aesthetic effects in tourism advertising.

Eye-tracking technology offers a powerful tool for investigating visual aesthetic and attention mechanisms [2,5,6,7]. By recording the location and duration of fixations, one can objectively understand viewers’ attention distribution patterns when browsing ads [8]. An empirical study has shown that the position and complexity of visual elements in ads significantly influence viewers’ gaze paths [9]. Observers’ eyes tend to first land on the most visually salient areas of an ad, such as a brightly highlighted image or a high-contrast headline. This viewing order reflects the human visual system’s automatic capture of attention by salient stimuli [10,11]. On the other hand, regarding the impact of visual complexity on consumer attention, findings show that the more abundant and cluttered the visual elements, the more dispersed viewers’ fixations become, and total fixation time is significantly extended [12,13]. Taken together, coupling eye-tracking with aesthetic evaluations clarifies how specific visual styles drive both attention and downstream judgments in tourism advertising.

### 2.2. Tourism Intention and Advertising Effect Theory

Tourism intention—the subjective likelihood of visiting a destination—arises from cognitive, affective, and social determinants [14]. The Theory of Planned Behavior (TPB) attributes intention to attitude, subjective norms, and perceived behavioral control; in tourism, liking for the destination, others’ opinions, and feasibility constraints jointly shape intention [15]. Many studies have examined how tourism advertisements increase tourism intention by influencing audiences’ attitudes and emotions [16,17]. For example, a study from Pakistan found that vivid and beautiful destination imagery can evoke positive emotional responses, thereby increasing tourists’ longing for that destination [18]. Similar findings were also found in the context of Chinese elements. A destination image with strong aesthetic appeal can give viewers a sense of “being there” and an immersive imagination, thus sparking a higher intention to visit [19].

Destination image theory further specifies the structure of intention formation: cognitive impressions, affective evaluations, and conative tendencies together form an image that predicts travel behavior [20], a pattern supported by meta-analytic evidence linking each dimension to behavioral intention [21]. Integrated analyses of numerous studies indicate that if tourism ads can successfully shape a positive cognitive and affective destination image, they will significantly enhance tourists’ willingness to visit.

On the other hand, specific visual styles and cultural symbols in advertisements can further influence tourism intention by shaping destination image. Studies have found that ads incorporating local cultural elements often enhance audiences’ emotional identification with and affinity for the destination. A study analyzed the cultural values of 48 New Zealand, 36 Indian, and 46 Chinese destinations and found significant differences among the three countries [22]. The study further found that cultural information on tourism websites affects travel intentions, which is related to the tourists’ own cultural background. The Consumer Aesthetic Value Model posits that consumers’ emotional, symbolic, and aesthetic perceptions of a product or experience collectively influence their overall evaluation and behavioral tendencies [23]. In the context of tourism advertising, the aesthetic pleasure and cultural meaning conveyed by an ad enhance viewers’ perceived value of the destination, which in turn translates into a stronger desire to travel. Research also supports this: tourism ads with high aesthetic quality and cultural connotations can simultaneously increase viewers’ aesthetic enjoyment and intensify their longing and curiosity for the destination [1].

### 2.3. Visual Style, Cultural Preference, and Perceptual Mechanisms

Visual style carries cultural meaning in addition to formal features. Cultural Schema Theory posits that background knowledge shapes how people interpret visual information [24]. When viewers encounter culturally marked symbols (e.g., traditional Chinese ink aesthetics—blank space, atmosphere), matching schemas are activated, fostering resonance and favorable attitudes toward the ad and destination [19]. The influence of cultural schemas is applicable to people from different cultural backgrounds or regions [25], such as Mediterranean countries [26], India [27], Malaysia, etc. [28].

Cross-cultural evidence corroborates schema effects: when tourists from different cultures view the same travel images, their focal attention and scanpaths differ systematically [29]. Broadly, Eastern viewers tend to prioritize contextual relations and atmosphere, whereas Western viewers more often prioritize focal objects and explicit details—differences that reflect culturally shaped perceptual preferences. Thus, cultural background can modulate both attention allocation and aesthetic preference in response to a given style.

Beyond identification, visual style also shapes perceived value and memory. Artistic style and cultural symbolism influence attention routes and recall [30]. In brochures, images and text attract attention differently, and viewing time relates to perceived novelty [31]. Integrating Cultural Schema Theory with consumer value perspectives helps explain how style affects immediate attention and affect as well as longer-term memory and attitudes.

### 2.4. Destination Context (Natural vs. Urban) as a Theoretical Moderator

A key omission in prior work is an explicit rationale linking destination context (natural vs. urban) to the perception of ink styles. Environmental psychology provides such a rationale. Attention Restoration Theory argues that natural environments afford “soft fascination,” coherence, and restoration, eliciting low arousal and harmonious affect [32]; Psychoevolutionary Theory similarly finds that unthreatening natural scenes promote stress recovery [33]. Urban scenes, by contrast, often present higher visual density, angular geometry, and external stimulation, heightening arousal and novelty seeking.

Mapping these contextual tendencies onto aesthetic dimensions yields specific predictions. When the formal vocabulary of an ad aligns with the affective affordances of a scene, processing should be more fluent and evaluations more positive. Thus, traditional ink—with its emphasis on harmony, subtle gradation, and negative space—should exhibit stronger congruity with natural landscapes, amplifying tranquility and coherence cues. Modern ink—with higher contrast, fragmented geometry, or dynamic composition—should exhibit stronger congruity with urban destinations, echoing energy, modernity, and innovation. Under a fluency/congruity account, such style–context fit should increase attention to diagnostic regions, lower TTFF to focal elements, and elevate aesthetic evaluation and travel intention; misfit should reduce fluency and dampen persuasion unless reframed as creative novelty. This moderator logic directly motivates the interaction tests in our study.

## 3. Materials and Methods

### 3.1. Participants

Young people are a very important group in modern tourism and are also an important target of tourism advertising exposure. This study targets this group and adopts a convenient abstract method to recruit participants from colleges and universities. A total of 80 undergraduates from universities in mainland China (42 males, 38 females; ages 18–26, M = 21.3, SD = 1.9) were recruited as participants. All participants had no professional training in art or advertising design, had normal or corrected-to-normal vision, and reported no history of neurological or visual-cognitive disorders. Prior to the experiment, each participant signed a written informed consent form.

To control for the influence of prior travel experience on ad evaluations, we asked participants during recruitment to fill out a basic information questionnaire indicating whether they had lived or spent an extended period during the past year in any of the four destinations involved in this study (Huangshan, Jiuzhaigou, Shanghai, Shenzhen). If a participant was very familiar with any target destination in the past year, they were excluded to avoid such prior familiarity influencing their responses to the ads.

Participants were then randomly assigned by a computer program to one of two experimental conditions: Group A (n = 40) viewed four tourism ad images in a traditional ink painting style, while Group B (n = 40) viewed four images in a modern ink painting style. Each participant completed the browsing and evaluation tasks for 4 ad images. After the experiment, we randomly selected 5 participants from each group for follow-up interviews. All participants received a remuneration of RMB 50 upon completing the experiment.

### 3.2. Experimental Design

We employed a 2 (ad ink painting style: traditional vs. modern) × 2 (destination type: natural landscape vs. urban scenery) mixed-factor experimental design. Ink painting style was a between-subjects factor, with each participant exposed to ads of only one style (traditional or modern). Destination type was a within-subjects factor, with each participant viewing ads of both content types (2 images of natural landscapes and 2 images of urban scenes). The combination of the two factors produced 4 experimental conditions, each corresponding to one specific ad image.

The dependent variables fell into two categories: (1) Objective visual attention metrics obtained via eye-tracking: total fixation duration (TFD). TFD refers to the cumulative fixation time on a given area of interest (AOI) by a participant, reflecting that area’s attentional attraction. (2) Subjective evaluation scores, comprising two dimensions: aesthetic preference and tourism intention. Both were measured using 7-point Likert scales (1 = strongly disagree, 7 = strongly agree). Each dimension consisted of 4 items, and the mean of the items’ scores was taken as the overall score for that dimension.

### 3.3. Stimuli Materials

**Figure 1:**
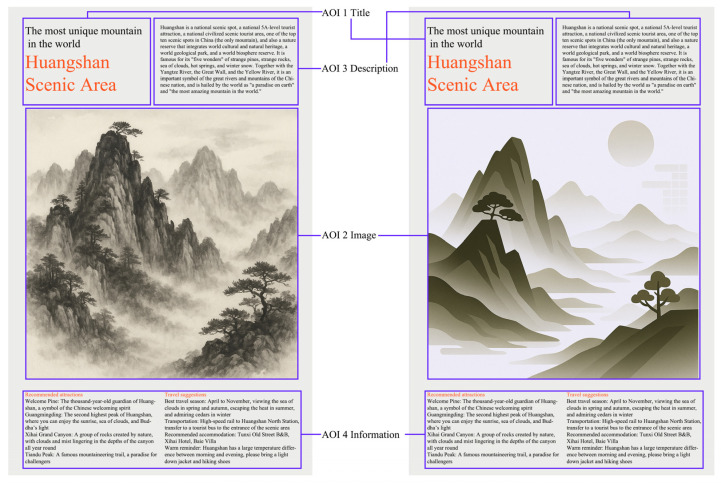
Ad image of Huangshan Scenic Area in traditional ink painting style and modern ink painting style (text has been translated into English).

The stimulus materials were custom-designed by the research team and consisted of 8 static tourism advertisement images covering 4 well-known tourist destinations in China: Huangshan, Jiuzhaigou, Shanghai, and Shenzhen. These four locations were chosen because they represent archetypal natural scenic sites (Huangshan, Jiuzhaigou) and modern urban landscapes (Shanghai, Shenzhen), providing a contrast in content. For each destination, two advertisement images were created: one using a traditional ink painting style and one using a modern ink painting style, forming four pairs of images. In each pair, the promotional content was kept consistent (same location) while the visual presentation style differed. Figure 1 shows an example of an ad image.

The image creation process was as follows: first, we collected representative photographs of each destination, ensuring they depicted the site’s iconic landscapes or cityscapes. We provided a large set of photographs for each destination to a generative AI image tool (a plugin built on the DALL-E 3 model in ChatGPT 4) and prompted it to output images in traditional ink painting and modern ink painting styles. After several iterative rounds, we selected two ink-style images of different styles for each destination. We then used digital image editing software (Adobe Photoshop CC) to make fine adjustments and add textual descriptions; the text was sourced from the destinations’ official websites. The traditional ink-style images emphasize expressive effect and ink texture, employing techniques from Chinese painting such as reserved blank space, splashed ink, and ink outlines; the overall picture appears as if painted with ink on rice paper, presenting an elegant ink-wash atmosphere. The modern ink-style images, in contrast, incorporate contemporary design elements such as flat graphic composition, geometric lines, colorful ink gradients, and digital brush effects, so that the image retains the freehand spirit of ink art while also exhibiting a stylish modern visual impression. Three experts in visual design and three experts in tourism independently evaluated the stimuli. The evaluation focused on two aspects: (1) whether the images effectively represented the regional characteristics of the tourist destinations, and (2) whether the visual style of the images was appropriately maintained. Based on the experts’ feedback, the stimuli were confirmed to reflect the unique features of the destinations, and the styles of traditional and modern Chinese ink paintings were successfully preserved.

To ensure comparability of image content across conditions, we standardized the format and layout of the 8 images. All images were adjusted to 1536 × 2372 pixels in size and presented in a vertical poster orientation. In each layout, we defined 4 predetermined AOIs (Figure 1): the title area (containing the destination name or slogan), the main image area (showcasing the primary landscape subject), the description text area (a short promotional blurb), and the information area (containing travel information such as suggestions). These four AOIs were kept uniform in relative size and position across all images to avoid layout differences influencing gaze behavior.

### 3.4. Instruments

Eye-tracking data were collected using Tobii Pro Fusion. The experiment was conducted in a quiet, softly lit laboratory. Participants sat approximately 60 cm from the display, and head movements were not restrained during the experiment to preserve a natural viewing state. Stimuli were presented on a 23-inch LCD monitor (1920 × 1080 resolution, 60 Hz refresh rate), with images displayed full-screen at the center against a neutral gray background to minimize visual distractions. The experimental procedure was controlled, and eye-tracking data were recorded using Tobii Pro Lab 1.102.

For subjective evaluations, we developed a questionnaire comprising two parts: aesthetic preference and tourism intention. After viewing each ad image, participants immediately completed this questionnaire, providing a subjective evaluation of the ad they had just seen. All items used 7-point Likert scales (1 = strongly disagree, 7 = strongly agree). The aesthetic preference scale contained 4 items, adapted from foundational research on visual design and aesthetic judgment [3,4]. Example items include: “I find this image visually attractive,” “The visual style of the image is aesthetically pleasing,” “The design elements in the image appear harmonious and well-coordinated,” and “This image presents a novel and interesting style.” The tourism intention scale also contained 4 items to assess the ad’s impact on participants’ interest in traveling and behavioral intention, adapted from classic measures in destination marketing research [14,34]. Example items include: “I would consider adding this destination to my future travel plans,” “This ad increased my interest in the destination,” “I want to learn more about this place,” and “If given the opportunity, I would prioritize visiting this destination.” In a pilot test, both scales showed good internal consistency (Cronbach’s α > 0.80).

Additionally, we designed a semi-structured interview outline to supplement the interpretation of the eye-tracking and survey results. The interview questions were centered on three core topics: (1) participants’ visual impressions and aesthetic evaluations of the different ink painting styles (traditional vs. modern); (2) the cultural connotations, style perceptions, and emotional connections conveyed by the images; and (3) the ads’ potential influence on participants’ desire to travel. All questions were open-ended, and the interviewer followed up with probing questions based on each interviewee’s responses to gain deeper cultural explanations and insights into individual cognition.

### 3.5. Procedures

**Figure 2:**
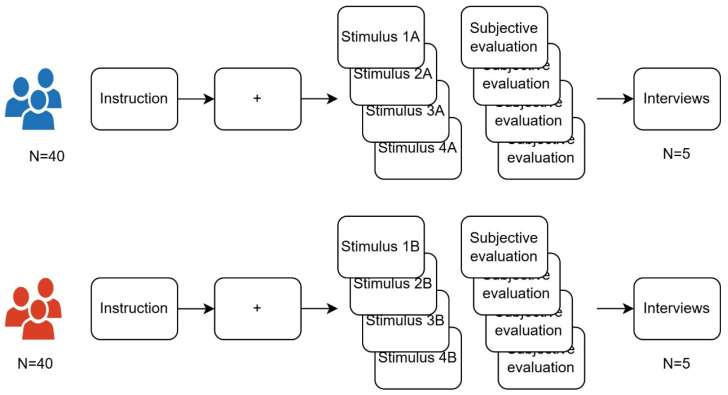
Experimental process.

Before the formal experiment began, the researcher briefly explained the study’s purpose and procedure to participants, emphasizing that this was an academic study with no right or wrong answers and that they should respond according to their true feelings. Participants then signed the informed consent form and filled out a basic information questionnaire to confirm they met the inclusion criteria. Group A corresponds to the traditional ink painting style, and Group B corresponds to the modern ink painting style. The experimental process is shown in Figure 2.

The experiment consisted of four trials, each with the following procedure: first, at the start of each trial, a black “+” fixation point was presented in the center of the screen for 1000 ms to calibrate the participant’s initial gaze position. Next, a tourism ad image was presented (in a traditional or modern ink style according to the participant’s group), and remained on screen for 20 s. During this time, participants were instructed to view the ad naturally, without any button presses or verbal responses. After 20 s, the screen automatically switched to the subjective evaluation questionnaire interface. Participants rated the ad they had just seen on four items for each of the aesthetic preference and tourism intention dimensions, then clicked submit to proceed to the next stimulus.

To control for any potential effects of presentation order, we randomized the order of the 4 images for each participant using a Latin square design. Each participant saw 2 natural landscape ads and 2 urban scenery ads, but the sequence (which type of image was seen first or last) varied by person. This design effectively balanced out any confounding effects that viewing order might have on gaze behavior or rating results.

After completing four rounds of ad viewing and questionnaires, the system indicated the end of the experiment. The researcher then invited some participants to take part in one-on-one semi-structured interviews, each lasting about 15–20 min. The interviewer asked questions based on the predefined outline and probed or clarified according to participants’ answers. The entire experimental session lasted about 30 min per participant. All experimental procedures strictly followed the research protocol and were supervised by dedicated personnel to ensure consistency and data validity.

### 3.6. Data Analysis

The data analysis of this study comprised quantitative and qualitative parts, corresponding, respectively, to the experimental data from eye-tracking and questionnaires, and the textual data from interviews. A total of 96 sets of eye-tracking data were collected. Among them, 16 were excluded due to a sampling rate below 90%. As a result, data from 80 participants were included in the final analysis.

For the quantitative analysis, we used SPSS 26.0 for data processing and statistical testing. We conducted 2 × 2 mixed-design analyses of variance (ANOVAs) for each dependent variable, with ink painting style as a between-subjects factor and destination type as a within-subjects factor. For the eye-tracking data, we calculated each participant’s average TFD on the image region (AOI 2) and the text region (combined AOIs 1 + 3 + 4) of each ad, as indicators of visual attention, and then performed two-factor ANOVAs comparing between groups (traditional vs. modern) and within groups (nature vs. city). For the subjective evaluation data, we computed each participant’s mean aesthetic score and intention score across images, and similarly entered these into the mixed-design ANOVA model to test for main effects and interactions. All statistical tests were two-tailed with a significance level of α = 0.05.

For the qualitative analysis, we adopted a thematic analysis approach to code and synthesize the interview data. Specifically, two trained research assistants independently read the interview transcripts and used open coding to mark important information in the text, initially identifying a number of conceptual labels. The research team then held a discussion to compare the results of the two coders and reconcile any coding discrepancies, thereby improving consistency and reliability [35]. After integrating perspectives, we merged similar codes and distilled them into several themes (categories), each theme representing a common viewpoint or experience among participants on a particular aspect. These themes were then used to explain the trends and differences observed in the quantitative data, helping us to understand the cognitive and emotional mechanisms behind the effects of different ad styles.

## 4. Results

### 4.1. Quantitative Results

#### 4.1.1. Aesthetic Evaluation

**Figure 3:**
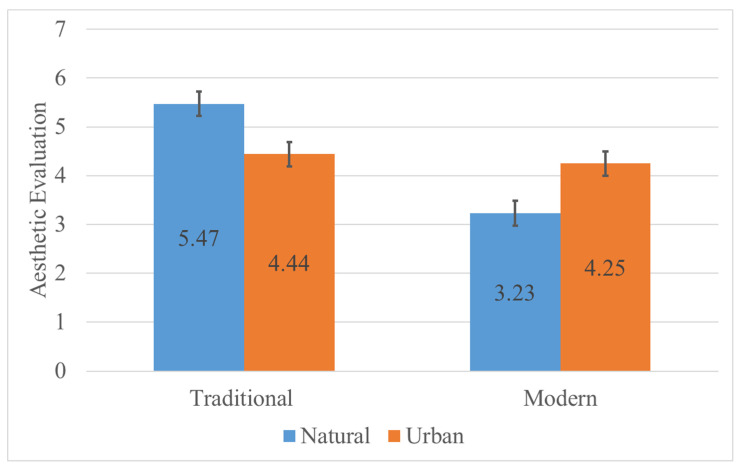
Interaction between style and destination in aesthetic evaluation. The aesthetic evaluation of traditional is higher in Natural, while the aesthetic evaluation of urban is higher in Modern.

Descriptive statistics for aesthetic evaluation are shown in Table 1. There was a significant main effect of style, with the traditional style yielding significantly higher aesthetic scores than the modern style, F (1, 78) = 80.981, *p* < 0.001, η_p_^2^ = 0.982. However, the main effect of destination type was not significant (*p* = 0.968). There was a significant interaction between style and destination type (Figure 3), F (1, 78) = 176.592, *p* < 0.001, η_p_^2^ = 0.694. Simple effects (Table 2) showed that in natural landscapes, the traditional style produced significantly higher scores than the modern style; under the traditional style condition, natural landscapes scored significantly higher than urban scenes; under the modern style condition, urban scenes scored significantly higher than natural landscapes (*p* < 0.05).

#### 4.1.2. Tourism Intention

**Figure 4:**
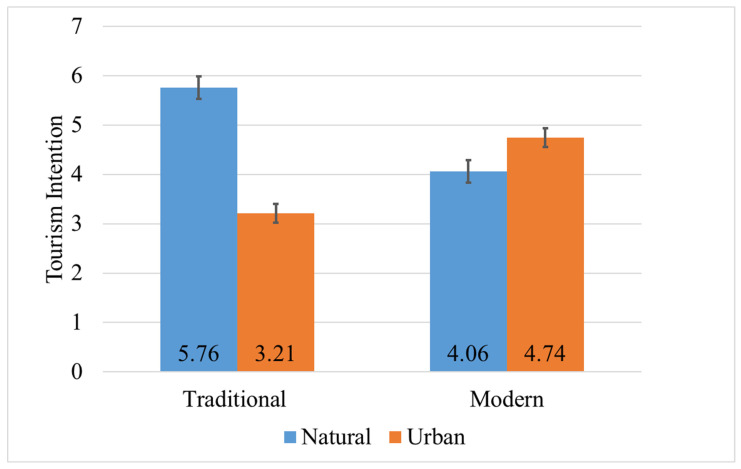
Interaction between style and destination in tourism intention. Among natural, traditional tourism, intention is higher, while among modern, urban tourism, intention is higher.

Descriptive statistics for tourism intention are shown in Table 3. There was no significant main effect of style (*p* = 0.410), but there was a significant main effect of destination type: natural landscapes scored significantly higher than urban scenes, F (1, 78) = 102.011, *p* < 0.001, η_p_^2^ = 0.567. The interaction between style and destination type was significant (Figure 4), F (1, 78) = 307.355, *p* < 0.001, η_p_^2^ = 0.798. Simple effects (Table 4) showed that in natural landscapes, the traditional style yielded significantly higher intention scores than the modern style; in urban scenes, the modern style yielded significantly higher scores than the traditional; under the traditional style, natural landscapes scored significantly higher than urban scenes; under the modern style, urban scenes scored significantly higher than natural landscapes (*p* < 0.05).

#### 4.1.3. Total Fixation Duration

**Figure 5:**
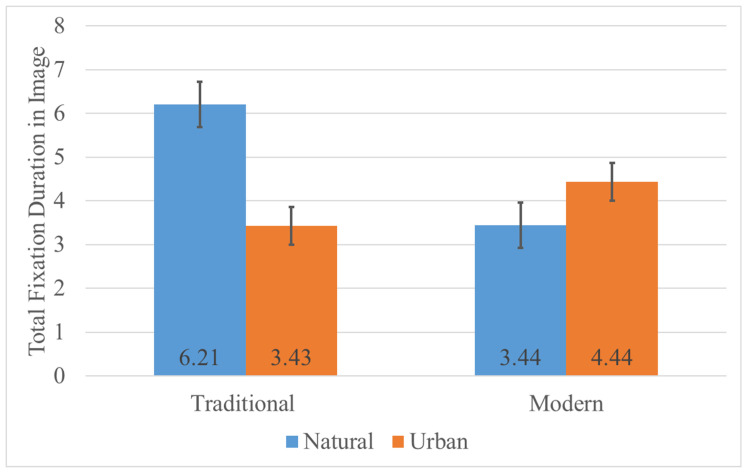
Interaction between style and destination in total fixation duration in image. The total fixation duration of traditional in natural is higher, while the total fixation duration of urban in modern is higher.

Descriptive statistics for total fixation duration are shown in Table 5. For the image regions, there was a significant main effect of style, with the traditional style producing a significantly longer TFD than the modern style, F (1, 78) = 15.058, *p* < 0.001, η_p_^2^ = 0.162, and a significant main effect of destination type, with natural landscapes having a significantly longer TFD than urban scenes, F (1, 78) = 21.336, *p* < 0.001, η_p_^2^ = 0.215. The interaction between style and destination type was significant (Figure 5), F (1, 78) = 97.261, *p* < 0.001, η^2^ = 0.555. Simple effects (Table 6) showed that for natural landscapes, TFD in the traditional style was significantly greater than in the modern style; for urban scenes, TFD in the modern style was significantly greater than in the traditional style; under the traditional style condition, natural landscapes had a significantly greater TFD than urban scenes; and under the modern style condition, urban scenes had a significantly greater TFD than natural landscapes (*p* < 0.05). For the text regions, there was a significant main effect of style, with the traditional style yielding a significantly longer TFD than the modern style, F (1, 78) = 53.682, *p* < 0.001, η_p_^2^ = 0.408, and a significant main effect of destination type, with urban scenes having a significantly longer TFD than natural landscapes, F (1, 78) = 228.484, *p* < 0.001, η_p_^2^ = 0.746. The interaction between style and destination type was not significant (*p* = 0.088).

### 4.2. Qualitative Results

In the qualitative interviews, participants shared a wealth of perspectives on their subjective experiences with the traditional and modern ink-style tourism ads. Through thematic analysis, we distilled the following common themes:

(1) Perception of Visual Style Characteristics

Participants clearly distinguished the two styles at first glance. Traditional ink was described as “elegant and serene… like a Chinese landscape painting” (P3), whereas modern ink was “bright, high-contrast, and instantly attention-grabbing” (P4). Several noted that modern compositions could feel “a bit cluttered,” demanding extra parsing, while traditional layouts appeared clearer and calmer. Overall, viewers recognized a contrast between restraint/clarity (traditional) and impact/energy (modern).

(2) Aesthetic Preference and Reasons

Preferences split between cultural familiarity and refined beauty (traditional) and novelty and trendiness (modern). As one participant put it, “I prefer the traditional ink style… it carries artistic charm and familiar culture” (P2). Others valued modern style as “creative and refreshing” (P7), yet some framed its appeal as stimulus-driven rather than emotionally deep—“cool, but with less meaning” (P9). The trade-off participants articulated was harmony/coherence versus novel stimulation.

(3) Feedback on Visual Elements

Viewers highlighted specific features guiding attention. Traditional ads’ brush strokes and blank space were “simple but enduring,” helping the focus “stay clear” (P1). Modern ads’ gradients, 3D effects, and digital composites increased interest and saliency but could be distracting when overused—“too many colors can be dazzling” (P8). These accounts underscore how saliency and complexity shape gaze allocation and processing effort.

(4) Emotional Connection and Cultural Meaning

Traditional style more readily evoked cultural pride, nostalgia, and comfort—“reminds me of traditional Chinese culture; it feels comforting” (P5)—and, for some, a brief sense of immersion, “as if inside a painting.” Modern style elicited curiosity and surprise, yet several viewers reported weaker cultural resonance: “cool, but with less cultural flavor and emotional resonance” (P10). Thus, traditional ads anchored meaning in shared cultural schemas, whereas modern ads emphasized experiential novelty.

(5) Impact on Tourism Intention

Both styles could raise willingness to visit when attractive. Traditional ink conveyed heritage and value—“deep cultural heritage; it makes me want to experience it in person” (P1)—while modern ink heightened curiosity—“trendy and different; it makes me want to see what the place is like” (P7). Participants also emphasized a boundary condition: ad style is supportive but not decisive—destination attributes ultimately determine intent (P4).

Interview evidence converges with the experiment: traditional ads’ higher aesthetic ratings align with reports of artistic mood and cultural identification; modern ads’ stronger initial pull but shallower emotional depth reflects comments on high saliency yet weaker cultural meaning. Together, findings support a mechanism whereby style-specific saliency/complexity influences attention, which relates to evaluation and intention, moderated by cultural interpretation.

## 5. Discussion

By integrating objective eye-tracking data with subjective questionnaire and interview feedback, this study comprehensively revealed the similarities and differences between traditional ink-style and modern ink-style tourism advertisements in terms of visual attention, aesthetic preference, and tourism intention, and we have sought to explain and discuss these findings from theoretical perspectives.

First, our results showed that the ink painting style of the advertisement had a significant effect on viewers’ visual attention distribution. The eye-tracking experiment indicated that the two styles differ in how they capture attention: overall, the traditional ink ads demonstrated stronger sustained attraction. We recorded that in a natural landscape context, viewers’ total fixation time on the traditional ink ad images was significantly longer than on the modern style ads, whereas in an urban context, the opposite was true (the modern style held a slight advantage). In other words, when the ad’s style is highly congruent with the type of scenery (e.g., traditional ink with mountains-and-water nature, modern ink with urban scenes), viewers linger longer on the image and invest more attention. This phenomenon of style–content congruence affecting attention can be explained from the perspective of visual attention theory and perceptual fluency [2,36]. When a visual style matches viewers’ existing aesthetic schemas and expectations, information processing is smoother, and viewers are more willing to repeatedly gaze and savor its details [3]. Conversely, if the style and content are incongruent, it may lead to attention being dispersed or processing being disrupted, thereby shortening viewing duration.

A potential mediator is narrative coherence [37]. Beyond attentional capture, we propose that narrative coherence—the perceived logical fit between an ad’s visual form and the destination story it conveys—acts as a mediator linking style–content congruence to evaluation and intention. When visual style aligns with the semantic and affective affordances of the scene (e.g., traditional ink with natural landscapes; modern ink with urban settings), viewers experience a clearer storyline and higher processing fluency, which together support aesthetic appraisal [3,38]. Qualitative reports that traditional layouts felt “not cluttered” and “the focus is clear” suggest greater perceived coherence, while comments about modern layouts being “a bit cluttered” indicate potential coherence loss. At the process level, such coherence should manifest as more ordered scanpaths and longer coherent dwell episodes, beyond mere first-glance saliency [2,39,40]. Future work could model coherence as a measured mediator (e.g., a 3–4 item scale on storyline clarity and visual–semantic fit) and test indirect effects while controlling for stimulus saliency.

Secondly, regarding aesthetic evaluation and preference, the quantitative results showed that overall, the traditional ink-style ads received higher aesthetic ratings, a superiority especially pronounced in the natural landscape scenario. Audiences’ preference for the traditional style largely stemmed from the cultural resonance and aesthetic pleasure it evoked. According to the five-stage aesthetic processing model, cultural familiarity and schema activation influence the cognitive evaluation stage of aesthetic experience [3]. For Chinese viewers, the traditional ink painting style activates deep cultural schemas [24], causing them to feel a sense of familiarity and a positive aesthetic bias early in perceptual processing. This perceptual fluency and familiarity-induced pleasantness explains why the traditional style ads were more readily accepted and liked—as reflected in many participants’ comments in interviews that the ads felt “comfortable” and “culturally rich” [36,41]. At the same time, it must be noted that the novel modern ink style is not without appeal. Although its average aesthetic rating was slightly lower than the traditional style, the modern style’s creative presentation won some viewers’ favor, and notably, the difference in aesthetic ratings between the two styles narrowed for city-themed ads. This can be understood via the dual-dimension theory of classical vs. expressive aesthetics [4]: the traditional ink style emphasizes order, harmony, and other classical aesthetic dimensions, and thus is better suited to conveying the tranquil beauty of natural landscapes. Meanwhile, the modern ink style emphasizes novelty, creativity, and other expressive aesthetic dimensions, better showcasing the vibrancy and fashionable feel of urban scenes. When aesthetics are matched with content, viewers’ subjective evaluations of the ad also improve accordingly [42,43]. This was evident in our results: the modern ink style performed relatively better in urban landscape ads than it did in nature landscape ads, indicating that expressive aesthetics are more appreciated in an urban context.

A potential mediator is perceived authenticity. The interviews repeatedly referenced “cultural richness,” “familiar culture,” and doubts about “less cultural flavor,” pointing to perceived authenticity as another mediator between style–content congruence and persuasive outcomes. Congruent pairings likely signal authenticity—that the ad’s look genuinely belongs to the place—thereby increasing credibility and warmth and strengthening the affective and symbolic components of destination image that, in turn, drive intention [23,44,45]. In our data, traditional ink in natural contexts appeared to heighten authenticity (e.g., “deep cultural heritage”), whereas modern ink sometimes reduced it for heritage-oriented scenes, even if it boosted novelty. Measuring authenticity (e.g., “the ad feels genuine/appropriate for this destination”) would allow a formal mediation test and clarify why equally attention-grabbing designs diverge in downstream motivation.

Lastly, the findings on tourism intention support the positive effect of aesthetic experience on attitudes and behavioral tendencies. Overall, the traditional ink ads, by enhancing viewers’ aesthetic affinity for and cultural identification with the destination, led to a stronger tendency toward travel intention. This is consistent with the theory of consumer aesthetic value: the emotional, symbolic, and aesthetic value viewers derive from an ad enhances their overall evaluation of the destination and thereby strengthens their behavioral intentions [23]. Our survey data showed that the aesthetic pleasure and cultural closeness evoked by the traditional style ads indeed translated into higher destination desirability scores. The qualitative interviews further corroborated this trend: several participants plainly stated that the traditional ink ads sparked the idea of visiting the destination, feeling that “the scenery has more cultural story, making it worth a trip” (P1, P5). Evidently, when an ad successfully conveys a destination’s aesthetic value and cultural meaning, audiences are more likely to feel longing and curiosity and be willing to embark on a journey [46]. By contrast, the effect of the modern ink ads on boosting tourism intention was somewhat limited. On one hand, their novel design piqued the interest of younger viewers and led to discussion, possibly aiding the destination’s word-of-mouth spread; but on the other hand, because some viewers doubted the ads’ authenticity and cultural substance, the modern style might not significantly strengthen the motivation to travel. As some interview comments pointed out, the curiosity sparked by visual creativity needs to be converted into trust in and yearning for the destination, which will influence travel decisions [44,45]. This suggests that the impact of ad style on tourism intention is indirect and conditional: only when the aesthetic and emotional responses elicited by the style successfully enhance the destination’s image will a positive effect on intention occur [47,48]. This conclusion aligns with the fundamental principle of the destination image model that cognitive, affective, and conative (intentional) components of image jointly predict travel intentions [20].

Building on the above analysis, our findings can be further interpreted from multiple theoretical perspectives. First, Cultural Schema Theory [24] was strongly validated in the context of this study. Traditional ink painting, as a classic symbol of Chinese culture, can rapidly activate domestic audiences’ cultural schemas, bringing cognitive familiarity and emotional identification [49]. This process reduced viewers’ difficulty in understanding the ad information and increased the content’s credibility and intimacy. This is the deep reason we observed that the traditional style ads held an advantage in both subjective evaluations and attention investment. In the field of cross-cultural communication, research has shown that tourists of different cultural backgrounds exhibit systematic differences in eye movement patterns when viewing the same ad [29], due to perceptual preference differences stemming from different cultural schemas. Our results are consistent with this, underscoring the role of cultural background in visual information processing: for viewers with Chinese cultural schemas, the traditional ink style fits their cognitive framework better, thus eliciting more positive responses [50,51].

Secondly, from the perspective of visual attention theory [2], our study enriches the understanding of attention allocation mechanisms in advertising. Visual attention theory proposes that consumers’ visual attention is jointly determined by stimulus-driven factors and goal-directed factors. Our eye-tracking results indicate that the traditional ink ads perform better in goal-directed sustained attention, with viewers repeatedly fixating because they have a greater interest in and preference for the content. This suggests that marketing communications practice should not single-mindedly pursue “instant eye-catch,” but also consider how to sustain viewers’ attention [52]. For tourism ads, using highly salient elements (e.g., bright colors, clever creativity) can increase immediate attention, but if the goal is for viewers to gain a deeper understanding and a lasting impression, the design must also incorporate content that resonates with viewers and prompts reflection [39,40]. For example, in our study, although the traditional ink style was not as bright as the modern style, its cultural imagery caused viewers to stay longer, gazing and savoring. This finding aligns with the advertising concepts of “stopping power” and “holding power”: an ideal ad should not only attract viewers to stop, but also make them willing to linger and appreciate [53]. Therefore, advertisers should consider both the eye-catching salience and the meaningfulness of visual elements to achieve better communication results.

Thirdly, in terms of aesthetic psychology and value perception, our study supports and extends existing theoretical frameworks. The aesthetic processing model emphasizes the role of perceptual familiarity and cognitive interpretation in forming aesthetic judgments [3]. Our results show that the traditional cultural style, by aligning with viewers’ aesthetic schemas, led to a smoother cognitive interpretation process and enhanced aesthetic pleasure, whereas the modern style required viewers to make more cognitive adjustments and adaptations, slightly affecting initial evaluations. This is consistent with the model’s predicted “prototype effect”: when an artistic stimulus is close to the viewer’s internal prototype or paradigm, it is more easily liked; conversely, innovation that deviates too far from the prototype may cause discomfort or a reserved attitude at the initial stage, even though novelty also brings some positive stimulation. It is noteworthy that the discussion of consumer value offers a broader perspective for understanding this phenomenon [23]. It points out that consumers’ evaluation of an experience is derived from a synthesis of multi-dimensional value perceptions, including functional, emotional, symbolic, and aesthetic aspects. In our context, the traditional ink ads simultaneously satisfied viewers’ aesthetic value (pleasurable artistic enjoyment), emotional value (evoking cultural pride and resonance), and symbolic value (embodying the destination’s cultural symbolism) [54,55], thus receiving a higher overall evaluation. In contrast, the modern ink ads mainly stimulated novel and interesting sensory excitement (a part of emotional value) but were somewhat lacking in symbolic meaning and emotional depth; hence, their overall evaluation was slightly lower. This corroborates the view of the Consumer Aesthetic Value model: only when an ad creates value on the aesthetic, emotional, and symbolic levels will audiences give the most positive feedback [3]. For tourism ads, the ability to balance creative expression with cultural substance is critical to their success.

Finally, our findings have direct implications for tourism destination marketing practice. On the one hand, the results indicate that incorporating indigenous cultural-artistic elements into tourism ads is an effective strategy. The traditional ink style significantly enhanced local audiences’ liking and interest in the ads, suggesting that a visual style with cultural recognizability helps boost a destination’s appeal and differentiated image. This aligns with prior research emphasizing cultural proximity: when marketing messages fit the target audience’s cultural background and aesthetic preferences, they more easily gain acceptance and trust [36,41]. Therefore, tourism destinations—especially those with rich historical and cultural heritage—should skillfully utilize traditional aesthetic elements in advertising to tell stories and convey emotion, thereby standing out in a homogenized marketing environment. On the other hand, we also observed that a modern creative style has advantages in attracting younger demographics [56]. For market segments seeking fashion and novelty, appropriately using modern ink or other innovative styles can increase ad attention and shareability. In practice, marketers might consider a context-matching strategy: select an appropriate visual style based on the type of destination and the target clientele [57]. For example, ads themed around cultural relics or natural landscapes could focus on a traditional aesthetic style to highlight their profound meaning, whereas ads for modern urban or tech-themed parks could incorporate modern artistic styles to showcase creativity and vitality. Meanwhile, caution should be taken to avoid relying excessively on visual spectacle while neglecting the conveyance of the destination’s core information. Whatever style is used, the ad content must clearly present the destination’s distinctive highlights and resonate with the audience’s travel motivations. Only by organically combining form and content can an advertisement be both “attractive” and “effective,” truly converting audience interest into action.

Despite the meaningful findings, several limitations merit caution and point to avenues for future work. First, our participants were young university students, a group typically more open to novelty; preferences for traditional versus modern styles may differ among older or international audiences. Future studies should broaden demographics and cultural backgrounds to test generalizability. Second, our stimuli focused exclusively on Chinese ink aesthetics, narrowing style diversity. Extending future studies to additional cultural art styles (e.g., oil painting, manga/comic, watercolor) would clarify whether the observed mechanisms generalize across visual traditions. Third, we began from four base photographs—two natural (Huangshan, Jiuzhaigou) and two urban (Shanghai, Shenzhen)—and rendered traditional/modern ink variants. Although this ensured a clean style × context manipulation, the small item sample raises external validity concerns and potential item-specific effects. Future work should (a) adopt broader item sampling (multiple destinations per category with intra-category diversity); (b) use mixed-effects models treating subjects and stimuli as crossed random factors; and (c) pretest and, where possible, equate low-level image properties (e.g., luminance, colorfulness, edge/texture density, visual complexity) while also collecting perceived congruence/coherence/authenticity ratings to partition alternative explanations. Fourth, the qualitative analysis relied on a random subsample of 10 interviewees out of 80, which may limit transferability and under-represent minority viewpoints. Increasing interview size, adopting stratified purposive sampling (e.g., by age, gender, art literacy, novelty seeking), documenting saturation, conducting member checking, reporting inter-coder reliability for a preregistered codebook, and complementing manual coding with computer-assisted text analysis would bolster credibility. Fifth, we used a single, laboratory-based exposure and did not track longer-term outcomes. Follow-ups (delayed recall, word-of-mouth, revisit intention) and field A/B tests would speak to durability and real-world impact. Sixth, although our discussion interprets several effects through cultural schemas, we assessed these processes qualitatively (interviews) rather than quantitatively via structured scales. As a result, we cannot formally test mediation by schema-related constructs. Future studies should include validated multi-item measures (e.g., cultural identification/familiarity with ink aesthetics, art literacy, need for cognition) and perceived style–content congruence, narrative coherence, and authenticity, enabling process-level mediation models that link style–context fit to evaluation and intention through these intermediaries.

## 6. Conclusions

Through eye-tracking experiments and interview analysis, this study compared the differences between traditional ink-style and modern ink-style tourism advertisements in visual appeal, aesthetic evaluation, and tourism intention. We found that traditional ink ads, in a natural landscape context, elicited longer fixation times, higher aesthetic scores, and stronger cultural resonance; the modern ink style, by contrast, was more initially eye-catching in an urban context and suited to conveying a sense of fashion and innovation. The congruence between visual style and destination content significantly influenced audience responses. Audiences’ preference for the traditional style was primarily driven by cultural familiarity and artistic atmosphere, whereas the modern style relied on visual novelty to capture attention. The theoretical significance of this study lies in extending Cultural Schema Theory and the aesthetic processing model to the field of advertising communication, revealing how visual style influences audience evaluations and behavioral intentions through perceptual processing and cultural connection. Practically, the study suggests that tourism ads should match an appropriate visual style to the type of destination and characteristics of the audience in order to enhance communication effectiveness and conversion rates. Traditional aesthetics help reinforce cultural impressions, while modern creativity is effective at grabbing attention. The two approaches should be combined and adapted to the context. Future research can broaden sample diversity, style types, and long-term effect tracking to further verify the generalizability and sustainability of our conclusions.

## Figures and Tables

**Table 1:** Descriptive results of aesthetic evaluation.

Style	Destination					
	Natural		Urban		Total	
	M	SD	M	SD	M	SD
Traditional	5.47	0.50	4.44	0.75	4.96	0.82
Modern	3.23	0.81	4.25	0.68	3.74	0.90
Total	4.35	1.31	4.35	0.72	4.35	1.05

**Table 2:** Results of simple effects for aesthetic evaluation.

Variable	I	J	Mean Difference (I−J)	F	*p*	η_p_^2^
Natural	Traditional	Modern	2.238	221.109	<0.001	0.739
Urban	Traditional	Modern	0.194	1.462	0.230	0.0108
Traditional	Natural	Urban	1.025	88.837	<0.001	0.532
Modern	Natural	Urban	−1.019	87.757	<0.001	0.529

**Table 3:** Descriptive results of tourism intention.

Style	Destination					
	Natural		Urban		Total	
	M	SD	M	SD	M	SD
Traditional	5.76	0.48	3.21	0.75	4.48	1.43
Modern	4.06	0.57	4.74	0.63	4.40	0.69
Total	4.91	1.01	3.98	1.03	4.35	1.12

**Table 4:** Results of simple effects for tourism intention.

Variable	I	J	Mean Difference (I−J)	F	*p*	η_p_^2^
Natural	Traditional	Modern	1.706	208.286	<0.001	0.728
Urban	Traditional	Modern	−1.537	98.874	<0.001	0.559
Traditional	Natural	Urban	2.556	381.752	<0.001	0.830
Modern	Natural	Urban	−0.687	27.613	<0.001	0.261

**Table 5:** Descriptive results of total fixation duration.

AOI	Style	Destination					
		Natural		Urban		Total	
		M (s)	SD	M (s)	SD	M (s)	SD
Image	Traditional	6.21	1.60	3.43	1.03	4.82	1.94
	Modern	3.44	1.40	4.44	1.22	3.94	1.40
	Total	4.82	2.05	3.94	1.23	4.38	1.74
Text	Traditional	5.52	1.40	8.95	1.34	7.23	2.20
	Modern	4.41	1.14	7.13	1.20	5.77	1.80
	Total	4.96	1.38	8.04	1.56	6.50	2.13

**Table 6:** Results of simple effects for total fixation duration in image.

Variable	I	J	Mean Difference (I−J)	F	*p*	η_p_^2^
Natural	Traditional	Modern	2.777	68.175	<0.001	0.466
Urban	Traditional	Modern	−1.017	16.245	<0.001	0.172
Traditional	Natural	Urban	2.785	104.853	<0.001	0.573
Modern	Natural	Urban	−1.008	13.744	<0.001	0.150

## Data Availability

The raw data supporting the conclusions of this article will be made available by the authors on request.
